# Age at natural menopause and associated factors with early and late menopause among Chinese women in Zhejiang province: A cross-sectional study

**DOI:** 10.1371/journal.pone.0307402

**Published:** 2024-07-16

**Authors:** Jie Jiao, Jiajun Hao, Leying Hou, Zeyu Luo, Shiyi Shan, Yuehong Ding, Linjuan Ma, Yizhou Huang, Qian Ying, Feixue Wang, Jianhong Zhou, Yumei Ning, Peige Song, Ling Xu

**Affiliations:** 1 Zhejiang Maternal, Child and Reproductive Health Center, Hangzhou, Zhejiang, China; 2 School of Public Health and the Second Affiliated Hospital, Zhejiang University School of Medicine, Zhejiang University, Hangzhou, Zhejiang, China; 3 Women’s Hospital, Zhejiang University School of Medicine, Hangzhou, Zhejiang, China; 4 Institute of Basic Medicine and Cancer (IBMC), Zhejiang Cancer Hospital, Chinese Academy of Sciences, Hangzhou, Zhejiang, China; Hamadan University of Medical Sciences, School of Public Health, ISLAMIC REPUBLIC OF IRAN

## Abstract

**Objectives:**

Menopause is a significant life transition for women, impacting their physical and psychological health. The age at natural menopause (ANM) and its associated factors have differed by race and region. This study aimed to investigate ANM and associated factors of early and late menopause among Chinese women in Zhejiang province.

**Methods:**

A cross-sectional study was conducted using a multi-stage stratified cluster sampling method to recruit 8,006 women aged 40–69 years who had resided in Zhejiang province for over 6 months between July 2019 and December 2021. Self-reported ANM and sociodemographics, lifestyle behaviors, reproductive history, and health-related factors were collected using questionnaires in face-to-face surveys. ANM were categorized into three groups: early menopause (<45 years), normal menopause (45–54 years), and late menopause (≥55 years). Kaplan-Meier survival analysis was utilized to calculate the median ANM. Multivariable multinomial logistic regression was employed to explore the associated factors of early menopause and late menopause.

**Results:**

A total of 6,047 women aged 40–69 years were included for survival analysis, with 3,176 of them for the regression analysis. The overall median ANM was 51 years (Inter-quartile range [IQR]: 51–52). Women who were smokers (odds ratio [OR]:4.54, 95% confidence interval [CI]:1.6–12.84), had irregular menstrual cycles (OR:1.78, 95% CI:1.12–2.83) and hypertension (OR:1.55, 95% CI:1.09–2.21) had a higher odds ratio of early menopause, while central obesity (OR:1.33, 95% CI:1.03–1.73) and hyperlipidemia (OR:1.51, 95% CI:1.04–2.18) were factors associated with late menopause.

**Conclusions:**

This study revealed the associations between ANM and various factors among Chinese women. These factors included socio-demographic factors such as age; life behavior factors like current or prior smoking status; reproductive history factors such as irregular menstrual cycles, miscarriages, and breastfeeding; and health-related factors like central adiposity, hypertension, and hyperlipidemia. These findings provided a basis for understanding factors associated with ANM.

## Introduction

Natural menopause is defined as the cessation of menstruation for at least 12 consecutive months due to the natural loss of ovarian follicular function [1–3]. According to the World Health Organization (WHO), the age at which natural menopause typically occurs in women is 45–55 years, which is considered to be within the normal range [4,5]. Natural menopause occurring before 45 years is referred to as early menopause, while menopause occurring after 55 years is considered late menopause [1,2,6,7]. The timing of natural menopause can influence postmenopausal women’s health, with early menopause being associated with an increased risk of ischemic heart disease (IHD) [8], cardiovascular disease (CVD) [9], type 2 diabetes [10], cognitive decline [11], osteoporosis [12], cancer [3], and higher all-cause mortality [2]. On the other hand, late menopause has been linked to an increased risk of breast cancer [13], endometrial, and ovarian cancers [14,15]. Therefore, identifying the age at natural menopause (ANM) and factors associated with early and late menopause, especially those that are modifiable, is crucial for preventing some diseases and improving the quality of life of middle-aged women [16].

Menopause not only brings about biological changes but also has a significant impact on women’s emotional and psychological well-being. ANM, a complicated biological and sociocultural phenomenon [17], can be influenced by both genetic factors and non-genetic factors, including demographic factors, reproductive health, lifestyle, and health-related factors [17]. Generally, lower socioeconomic status (SES) [17], never use of oral contraceptives (OCs) [6], nulliparity [11], smoking [1], and being underweight [17] have been associated with early menopause. Conversely, higher education [18], breastfeeding history [19], use of OCs [6,17], multiparity [6,17], overweight, and obesity [20] have been associated with late menopause.

It is noteworthy that ANM varies across different racial and ethnic groups and geographic regions [17]. For instance, the median ANM in the United States, Australia, and Europe was higher than in Africa, Asia, and the Middle East [17]. Moreover, the associated factors of early and late menopause have been reported to vary across countries. For instance, alcohol consumption has been found to be associated with late menopause in Canada, the UK, and the US, but no such association has been found in Norway, Germany, and China [17]. Even within the same country, variations in ANM still exist, particularly in large countries with uneven social development levels, lifestyles, and geographic features like China, although these findings have not been consistent across all studies [21,22]. A recent survey conducted in northwestern China found the median ANM of women to be 48 years [21]. Another cross-sectional study in Jiangsu province found the median ANM of women was 50 years old [23]. To date, the ANM and associated factors of early and late menopause in one of the most developed provinces, Zhejiang, have rarely been reported. This study aimed to investigate the median ANM and associated factors of early and late menopause among women living in Zhejiang province.

## Materials and methods

### Study design and participants

The cross-sectional survey was conducted by the Zhejiang Maternal, Child and Reproductive Health Center and Women’s Hospital School of Medicine Zhejiang University between July 2019 and December 2021. The survey investigated the current health status and associated factors among women aged 40–69 years living in Zhejiang Province. A multi-stage stratified cluster sampling method was implemented to select survey sites with strata based on geographical administrative and economic development. Each stratum incorporated one urban and one rural site, resulting in a total of ten sites. In the selected sites, our research team, primarily composed of specialists from maternal and child health institutions, conducted health education sessions aimed at recruiting women for our survey. These health education sessions primarily focused on women’s health and provided detailed explanations of the study. Women aged 40–69 years who had lived in selected sites for more than 6 months were eligible for the survey. While those aged <40 years or ≥70 years, with artificial menopause (including hysterectomy, bilateral oophorectomy, and radiotherapy), mental disorders, history of malignancy, or history of immunosuppressant use were excluded. During our health education sessions, all the eligible women were invited to complete a self-administered questionnaire, facilitated by our fully trained field investigators. Out of the 8,006 individuals initially recruited, 990 (12.36%) were excluded due to not meeting the inclusion criteria, and 6,047 (75.52%) were found eligible for the study.

Data on demographic and socioeconomic factors, lifestyle and living environment factors, diet, psychological and emotional, adverse life events, reproductive history, gynecological disease history, menopause-related symptoms, and quality of life were collected using a combination of self-developed questions and standard questions adapted from validated instruments, specifically referring to the questionnaires from the China Kadoorie Biobank (CKB) study [24] and the Shanghai Women’s Health Study (SWHS) [25].

### Assessment of dependent variable

In this study, ANM was the primary outcome. Women’s menopausal status was assessed through the question “Have you experienced natural menopause?”. Women who had experienced cessation of menstruation for more than 12 consecutive months were asked to report the age at which this occurred (ANM) and the time elapsed since their last menstrual period (postmenopausal duration). According to the self-reported ANM, menopausal women were divided into three groups: early menopause (<45 years), normal menopause (45–54 years), and late menopause (≥55 years).

### Ascertainment of independent variables

Independent variables of interest, including sociodemographic characteristics, lifestyle behaviors, reproductive history, and health status, were chosen based on their relevance in studies on menopause and associated health outcomes. Sociodemographic characteristics included birth year, education (illiteracy, primary school, middle school, or high school and above), job type (professional and technical staff, or officers; workers, merchants, or service workers; farmers; others), marital status (couple, or single), hukou (urban, or rural), and average monthly income in 2018. The income levels were grouped into three categories: <¥3,000 (equivalent to less than $435.83 in 2018), ¥3,000-¥5,000 ($435.83-$726.38), or >¥5,000 (>$726.38).

Life behaviors considered were smoking status (never smoking, or any smoking), alcohol intake status (non-drinker or drinker), regular physical exercise, which was defined as at least twice a week and each time more than 30 minutes (no, or regular physical exercise), and sleep quality (general, good, or poor).

Reproductive history included age at menarche, divided into three groups: early menarche (9–11 years), normal menarche (12–15 years), and late menarche (16-18years) [26], menstrual cycle length, gravidity, parity, number of miscarriages, lactation history, lifetime lactation duration, contraceptive history, oral contraceptive pills (OCP) use, and use of hormone replacement therapy.

Health status included the self-reported status of hypertension, diabetes, and hyperlipidemia, all of which were confirmed by medical diagnoses. Additionally, body mass index (BMI) was calculated as self-reported weight divided by the square of self-reported height, and it was categorized into 3 levels (underweight: <18.5 kg/m^2^, normal weight: 18.5–23.9 kg/m^2^, overweight and obesity: ≥24 kg/m^2^) [27]. Self-reported waist circumference (WC) was used as a measure of central adiposity. If the WC was over 85cm, it was defined as central obesity for women [28].

### Statistical analysis

All continuous variables were found to be highly skewed from tests for normal distribution and thus were reported as median with interquartile ranges (IQRs). The Kruskal-Wallis rank sum tests were utilized to calculate differences among the three groups of ANM. Categorical variables were presented as numbers with percentages (%), and differences were analyzed using Pearson’s Chi-squared (χ^2^) tests or Fisher’s exact test.

Kaplan-Meier survival analysis was used to calculate the median ANM. The follow-up time was from the woman’s birth (age 0 years) until the age at menopause or attained age at data collection (censoring).

Multivariable multinomial logistic regression was employed to assess the associations between the independent variables and the dependent variable. The dependent variable was categorized into three groups: early menopause, normal menopause, and late menopause, with normal menopause serving as the reference category. In our logistic regression model, we employed both backward and forward stepwise methods to determine the inclusion and retention of independent variables. The Akaike Information Criterion (AIC) was utilized to assess the fit of the model. Odds ratios (ORs) and 95% confidence intervals (CIs) of associated factors were estimated in the model.

All statistical analyses were conducted using R software (version 4.2.1). Statistical significance was considered to be *P*<0.05 with a two-sided, or 95% CIs of OR that did not cross 1.00.

### Ethics

The survey obtained ethical approval from the Medical Ethics Committee Ethical Review Committee of the Zhejiang Maternal, Child and Reproductive Health Center. All participants signed the written consent forms, except for illiterate individuals who gave oral consent.

## Results

**Fig 1:**
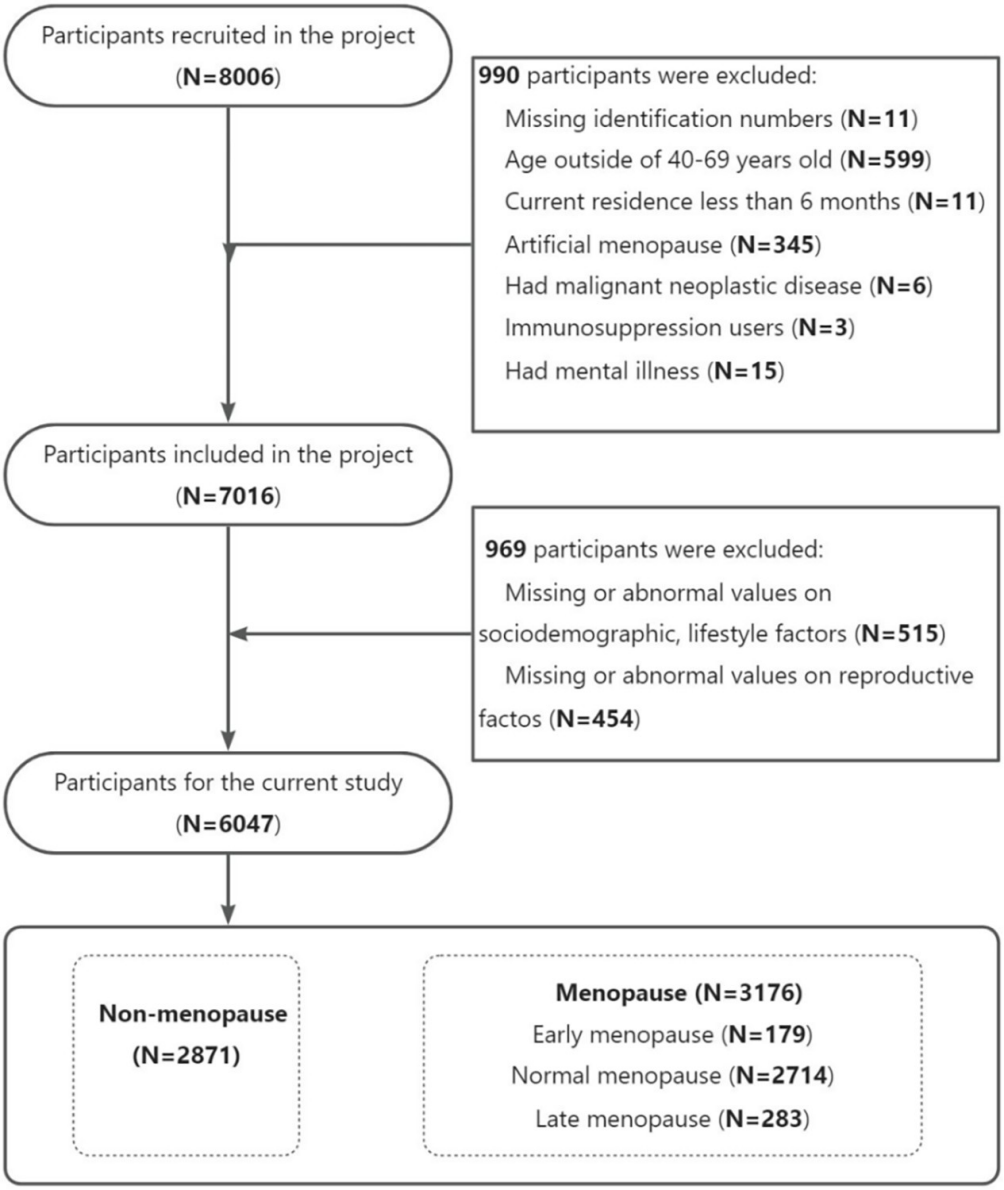
Flow chart of study participants selection.

Women who had missing identification numbers (n = 11), were aged outside of 40–69 years old (n = 599), had lived in the current residence for less than 6 months (n = 11), had artificial menopause (n = 345), suffered from malignant neoplastic disease (n = 6), were immunosuppression users (n = 3) or had a mental illness (n = 15) were excluded from the study for not meeting the inclusion criteria. Additionally, 515 women (6.43%) with missing or abnormal values (including height ≤ 50 cm or ≥200 cm, weight ≤20 kg or ≥ 400 kg, and WC ≤ 20 cm or ≥ 160 cm) in sociodemographic and lifestyle factors were removed from the study. 454 women (5.67%) with missing or abnormal values (including the age of menarche <9 or >18) in reproductive factors were also excluded from the study. After these exclusions, 6,047 women (75.52%) were deemed eligible and included in this study. Of them, 2,871 participants (35.86%) were non-menopausal women, and 3,176 (39.68%) were menopausal women (**Fig 1**).

The median age of all menopausal women at the time of the survey was 57 years (IQR: 54.0, 62.0). The median age at the time of the survey was 56 years (IQR: 47.5, 63.0) for early menopausal women, 57 years (IQR: 54.0, 62.0) for normal menopausal women, and 60 years (IQR: 57.0, 63.0) for late menopausal women (**Table 1**). The majority of menopausal women were partnered (94%) and from rural areas (64%). The highest educational level for early and late menopausal women was mostly primary school. Over 50% of them had less than ¥3,000 average monthly income in 2018. Statistically significant differences between the three menopausal groups can be seen in education and average monthly income in 2018. Compared to women who had a normal ANM or late ANM, early menopausal women were more likely to be smokers (2.8%), have irregular menstrual cycles (14%), never lactated (12%), and have never used OCP (24%) at the time of survey (all *P*<0.001). Women who had experienced late menopause were more likely to engage in regular physical exercise (66%), be overweight and obese (48%), exhibit central adiposity (42%), have hypertension (36%), and hyperlipidemia (15%) at the time of survey (all *P*<0.001). There were statistically significant differences in smoking status, regular physical exercise, menstrual cycle, lactation, lifetime lactation duration, contraceptive history (mainly referring to the use of contraceptive methods such as oral contraceptives), hormone replacement therapy, BMI, central adiposity, hypertension, and hyperlipidemia between early menopause, normal menopause, and late menopause (**Table 1**).

**Table 1:** Baseline characteristics of women in the study according to age at natural menopause.

Characteristic		Overall, N = 3,176	Early menopause (<45), N = 179	Normal menopause (45–54), N = 2,714	Late menopause (> = 55), N = 283	*P* value^#^	
**Age, years**		57.0 (54.0, 62.0)	56.0 (47.5, 63.0)	57.0 (54.0, 62.0)	60.0 (57.0, 63.0)		<0.001
**Education**							0.028
	Illiteracy	497 (16)	29 (16)	406 (15)	62 (22)		
	primary school	1,108 (35)	63 (35)	950 (35)	95 (34)		
	middle school	946 (30)	46 (26)	832 (31)	68 (24)		
	High school and above	625 (20)	41 (23)	526 (19)	58 (20)		
**Job**							0.146
	Professional and technical staff, officers	404 (13)	28 (16)	348 (13)	28 (9.9)		
	Workers, merchants, service workers	647 (20)	33 (18)	557 (21)	57 (20)		
	Farmers	1,196 (38)	77 (43)	1,017 (37)	102 (36)		
	Others	929 (29)	41 (23)	792 (29)	96 (34)		
**Marital status**							0.15
	Couple	3,001 (94)	167 (93)	2,573 (95)	261 (92)		
	Single	175 (5.5)	12 (6.7)	141 (5.2)	22 (7.8)		
**Hukou**							0.57
	Urban	1,138 (36)	58 (32)	975 (36)	105 (37)		
	Rural	2,038 (64)	121 (68)	1,739 (64)	178 (63)		
**Average monthly income in 2018, RMB (equivalent to US dollars($) in 2018)**							0.025
	<3000 ($435.83)	1,962 (62)	95 (53)	1,678 (62)	189 (67)		
	3000–5000 ($435.83- $726.38)	885 (28)	56 (31)	762 (28)	67 (24)		
	>5000 ($726.38)	329 (10)	28 (16)	274 (10)	27 (9.5)		
**Smoking status**							0.021
	Never smoking	3,149 (99)	174 (97)	2,695 (99)	280 (99)		
	Any smoking	27 (0.9)	5 (2.8)	19 (0.7)	3 (1.1)		
**Alcohol intake status**							0.571
	Non-drinker	2,916 (92)	161 (90)	2,497 (92)	258 (91)		
	Drinker	260 (8.2)	18 (10)	217 (8.0)	25 (8.8)		
**Regular physical exercise**							0.03
	No	1,268 (40)	81 (45)	1,092 (40)	95 (34)		
	Regular physical exercise	1,908 (60)	98 (55)	1,622 (60)	188 (66)		
**Sleep quality**							0.219
	General	1,277 (40)	77 (43)	1,097 (40)	103 (36)		
	Good	1,098 (35)	54 (30)	930 (34)	114 (40)		
	Poor	801 (25)	48 (27)	687 (25)	66 (23)		
**Menarche status**							NA
	Early menarche (9–11)	6 (0.2)	1 (0.6)	4 (0.1)	1 (0.4)		
	Normal menarche (12–15)	1,806 (57)	99 (55)	1,572 (58)	135 (48)		
	Late menarche (16–18)	1,364 (43)	79 (44)	1,138 (42)	147 (52)		
**Menstrual regularity**							0.022
	Regular (3-7days)	2,907 (92)	154 (86)	2,491 (92)	262 (93)		
	Irregular	269 (8.5)	25 (14)	223 (8.2)	21 (7.4)		
**Gravidity**							NA
	0	24 (0.8)	4 (2.2)	18 (0.7)	2 (0.7)		
	1	624 (20)	22 (12)	540 (20)	62 (22)		
	2	1,293 (41)	76 (42)	1,104 (41)	113 (40)		
	3	1,235 (39)	77 (43)	1,052 (39)	106 (37)		
**Parity**							NA
	0	38 (1.2)	7 (3.9)	27 (1.0)	4 (1.4)		
	1	1,642 (52)	77 (43)	1,431 (53)	134 (47)		
	2	1,496 (47)	95 (53)	1,256 (46)	145 (51)		
**Number of miscarriages**							0.67
	0	1,568 (49)	88 (49)	1,328 (49)	152 (54)		
	1	916 (29)	52 (29)	790 (29)	74 (26)		
	2	692 (22)	39 (22)	596 (22)	57 (20)		
**Lactation history**							0.004
	No	204 (6.4)	22 (12)	163 (6.0)	19 (6.7)		
	Lactation	2,972 (94)	157 (88)	2,551 (94)	264 (93)		
**Lifetime lactation duration, month**							0.03
	0	268 (8.4)	21 (12)	225 (8.3)	22 (7.8)		
	1–12	1,344 (42)	61 (34)	1,174 (43)	109 (39)		
	13–24	1,085 (34)	63 (35)	926 (34)	96 (34)		
	>24	479 (15)	34 (19)	389 (14)	56 (20)		
**Contraceptive history** *							0.017
	No	521 (16)	43 (24)	435 (16)	43 (15)		
	Contraceptive history	2,655 (84)	136 (76)	2,279 (84)	240 (85)		
**Oral contraceptive pills**							0.906
	No	2,916 (92)	165 (92)	2,493 (92)	258 (91)		
	Oral contraceptive pills	260 (8.2)	14 (7.8)	221 (8.1)	25 (8.8)		
**Hormone replacement therapy**							<0.001
	No	3,013 (95)	152 (85)	2,587 (95)	274 (97)		
	Hormone replacement therapy	163 (5.1)	27 (15)	127 (4.7)	9 (3.2)		
**BMI, kg/m2**							0.018
	Underweight (<18.5)	127 (4.0)	9 (5.0)	110 (4.1)	8 (2.8)		
	Normal weight (18.5–23.9)	1,801 (57)	106 (59)	1,557 (57)	138 (49)		
	Overweight and obesity (> = 24.0)	1,248 (39)	64 (36)	1,047 (39)	137 (48)		
**Central obesity, cm** **							0.002
	Non-central obesity (<85)	2,151 (68)	124 (69)	1,862 (69)	165 (58)		
	Central obesity(≥85)	1,025 (32)	55 (31)	852 (31)	118 (42)		
**Hypertension** **							<0.001
	No	2,320 (73)	125 (70)	2,014 (74)	181 (64)		
	Hypertension	856 (27)	54 (30)	700 (26)	102 (36)		
**Diabetes** **							0.065
	No	2,966 (93)	167 (93)	2,544 (94)	255 (90)		
	Diabetes	210 (6.6)	12 (6.7)	170 (6.3)	28 (9.9)		
**Hyperlipidemia** **							<0.001
	No	2,879 (91)	170 (95)	2,469 (91)	240 (85)		
	Hyperlipidemia	297 (9.4)	9 (5.0)	245 (9.0)	43 (15)		

Values are presented as median (M) with interquartile range (IQR) or number (N) with percent (%).

^#^ *P* values represent statistical measurement of comparing early, normal, late menopause group using Kruskal-Wallis rank sum test for continuous variables or Pearson’s Chi-squared test and Fisher’s exact test for categorical variables.

* This term referred to the main methods of contraception, which included oral contraceptives, topical contraceptives, condoms, intrauterine devices (IUDs), subdermal implants, contraceptive injections, coitus interruptus (withdrawal method), male sterilization, and female sterilization.

**The cases of central obesity, hypertension, diabetes, and hyperlipidemia referred to in this study are current conditions, not instances of these conditions prior to menopause.

**Fig 2:**
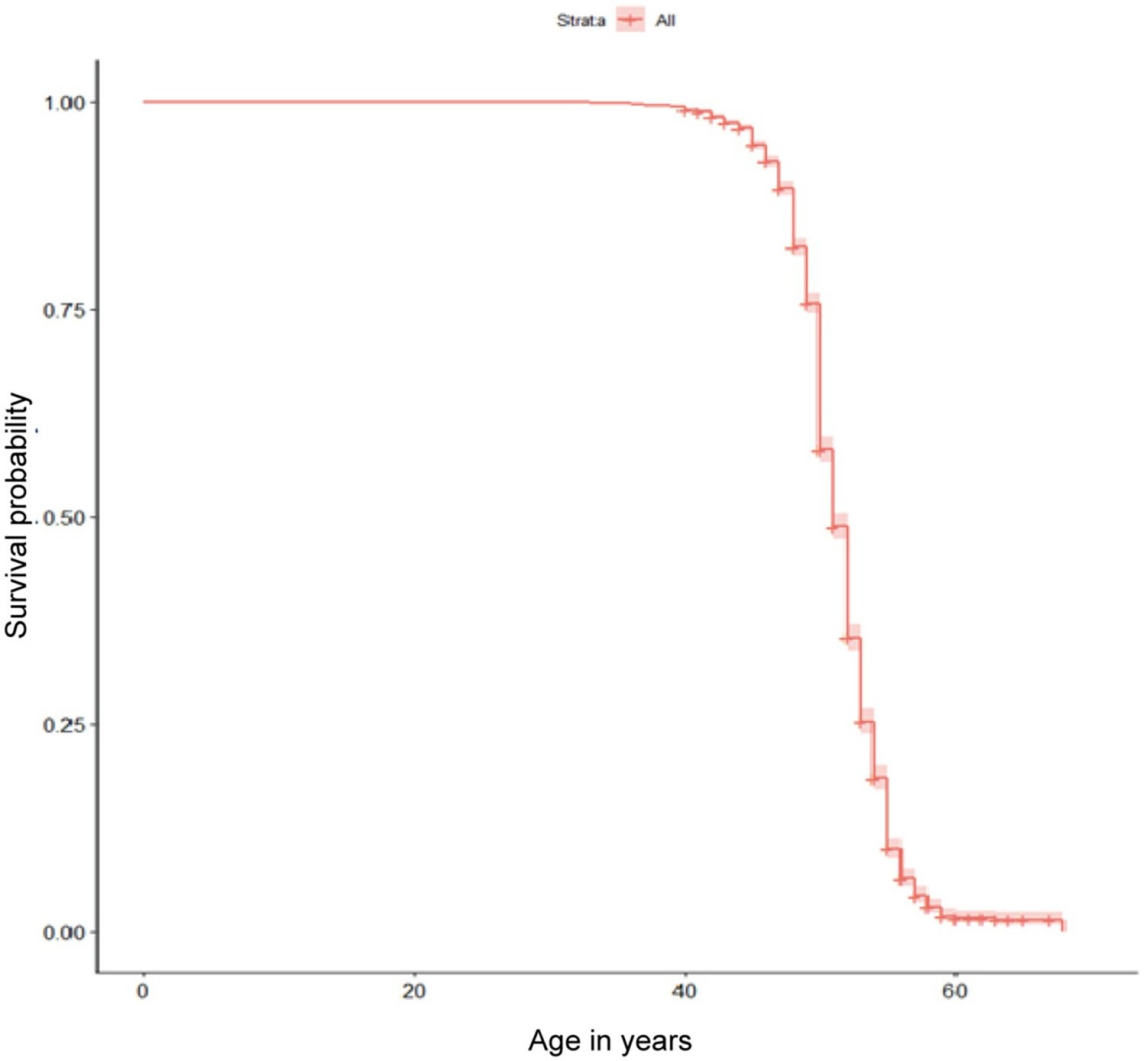
Kaplan-Meier cumulative estimates of age at natural menopause in study.

A survival curve of ANM for the 6,047 included women in this study revealed the median ANM was 51 (IQR: 51–52) years (**Fig 2)**. All independent variables were initially included in the multivariable multinomial logistic regression model for identifying factors associated with early and late menopause. A forward and backward stepwise selection process was employed to determine the associated factors, with normal menopause serving as the reference group (**Table 2**). From the adjusted logistic regression model, a history of smoking (OR: 4.54, 95% CI: 1.6–12.84), irregular menstrual cycles (OR: 1.78, 95% CI: 1.12–2.83), and having hypertension at the time of the survey (OR: 1.55, 95% CI: 1.09–2.21) were positively associated with early menopause. In contrast, older age at the time of the survey (OR: 0.93, 95% CI: 0.90–0.95), history of one miscarriage (OR: 0.58, 95% CI: 0.39–0.87), history of two miscarriages (OR: 0.40, 95% CI: 0.23–0.69) and history of breastfeeding (OR: 0.50, 95% CI: 0.30–0.85) were negatively associated with early menopause. In terms of late spontaneous menopause, compared with normal ANM, higher ORs were observed in women who were older at the time of the survey (OR: 1.10, 95% CI: 1.07–1.13), had central obesity (OR: 1.33, 95% CI: 1.03–1.73) and hyperlipidemia (OR: 1.51, 95% CI: 1.04–2.18).

**Table 2:** Multivariate logistic regression model of age at natural menopause and associated factors.

		Early menopause (40–44)	Late menopause (≥55)
		OR (95% CI)	OR (95% CI)
**Age, years**		0.93 (0.90, 0.95)	1.10 (1.07, 1.13)
**Smoking status**			
	Never smoking	Reference	Reference
	Any smoking	4.54 (1.6, 12.84)	1.62 (0.47, 5.6)
**Menstrual regularity**			
	Regular (3-7days)	Reference	Reference
	Irregular	1.78 (1.12, 2.83)	0.88 (0.55, 1.42)
**Gravidity**			
	0	Reference	Reference
	1	0.39 (0.11, 1.43)	1.26 (0.26, 6.01)
	2	0.94 (0.26, 3.35)	0.95 (0.20, 4.51)
	3	1.40 (0.38, 5.18)	0.79 (0.16, 3.84)
**Number of miscarriages**			
	0	Reference	Reference
	1	0.58 (0.39, 0.87)	1.05 (0.75, 1.47)
	2	0.40 (0.23, 0.69)	1.21 (0.78, 1.90)
**Lactation history**			
	No	Reference	Reference
	Lactation	0.50 (0.30, 0.85)	0.89 (0.53, 1.50)
**Hormone replacement therapy**			
	No	Reference	Reference
	Hormone replacement therapy	3.37 (2.06, 5.51)	1.05 (0.52, 2.14)
**Central obesity, cm** **			
	Non-central obesity (<85)	Reference	Reference
	Central obesity(≥85)	1.11 (0.79, 1.57)	1.33 (1.03, 1.73)
**Hypertension** **			
	No	Reference	Reference
	Hypertension	1.55 (1.09, 2.21)	1.24 (0.94, 1.63)
**Hyperlipidemia** **			
	No	Reference	Reference
	Hyperlipidemia	0.49 (0.25, 1.00)	1.51 (1.04, 2.18)

OR, odds ratio; CI, confidence interval. Multivariable multinomial logistic regression was estimated using backward and forward stepwise.

**The cases of central obesity, hypertension, diabetes, and hyperlipidemia referred to in this study are current conditions, not instances of these conditions prior to menopause.

## Discussion

Our study revealed that the median ANM among women in this study from Zhejiang province was approximately 51 years. Early menopause was associated with current or prior smoking status, a history of irregular menstrual cycles, and hypertension. Women who were older at the time of the survey, had central obesity and had hyperlipidemia were more likely to experience late menopause.

In our study, we observed a positive association between age at the time of the survey and late menopause in the adjusted logistic model, which is inconsistent with previous studies. Several prior studies have indicated that older women were more likely to report early menopause [29]. The exact mechanism through which current age influences ANM is still unclear, but it is likely related to economic improvements, changes in lifestyle, and enhancement in health status in more recent cohort [22,30]. This is particularly relevant for the older Chinese generation, who experienced the country’s great famine and may be at an increased risk of early menopause [17,31]. However, complete consensus has not been reached regarding this association.

Our results indicated that smoking was associated with an increased risk of early menopause, consistent with a substantial body of prior research showing that smoking was significantly associated with early menopause [21,23,32]. This phenomenon has been attributed to the negative effects of polycyclic aromatic hydrocarbons of cigarettes on ovarian aging, including lowering estrogen levels and damaging ovarian germ cells [33].

In our study, we found that reproductive history was closely related to ANM. Specifically, irregular menstruation was significantly positively associated with early menopause. Prolonged and continuous irregular menstruation may contribute to early menopause [34]. However, the precise role of irregular menstrual cycles in their association with ANM remains unclear and may be related to various physiological and modifiable factors. The hypothalamic-pituitary-ovarian (HPO) axis plays a key role in regulating the female reproductive system [35]. Functional hypothalamic amenorrhea (FHA) resulting from HPO dysregulation is the most important cause of irregular menstruation [34,36]. Higher BMI and stress are known risk factors for FHA, which can lead to irregular menstruation [37]. Furthermore, FHA can cause estrogen deficiency, potentially leading to early menopause [17,34]. A study based on Korea National Health and Nutrition Examination Survey found that higher BMI and stress had negative effects on menstrual cycle irregularity and early menopause [34]. Further studies are still needed to explore the precise association between irregular menstruation and early menopause.

In our study, we found that the history of miscarriages, which included both spontaneous miscarriages and induced abortions, was negatively associated with early menopause compared to women who did not report prior miscarriages. ANM has different associations with spontaneous miscarriages and induced abortions. A previous study indicated that induced abortions were associated with late ANM, while women with spontaneous miscarriages had a higher risk of early menopause [22]. The majority of participants in our study were affected by the one-child policy implemented between 1979 and 2015, leading to numerous induced abortions [38,39]. The birth cohort effect could be a major reason why our study showed the history of miscarriages was negatively associated with having early menopause [22]. Furthermore, women who had experienced the one-child policy usually had long-term contraception, which may potentially lead to a lower risk of early menopause [40–42]. The association between miscarriage and ANM is complex. Further studies need to focus on the association of natural miscarriages and induced abortions with ANM.

We also found that women with a lactation history were negatively associated with early menopause, which is consistent with some prior studies [1,11]. However, the mechanism of lactation’s effect on ANM is still vague. Some studies suggest that this might be attributed to breastfeeding, which could reduce ovulation and decrease aging. Consequently, women may experience a lower risk of early menopause [11,43].

Our study observed the associations between health status and ANM. Our results showed a positive association between current central adiposity and late menopause. This may be explained by the fact that women with central obesity usually have a high BMI, which is associated with increased estrogen production generation that could delay menopause [44,45]. It is noteworthy that the measurement of central adiposity in our study was based on self-reported WC. Considering that WC is not typically examined during health check-ups, the use of subjective and recollected WC data could potentially influence the observed association. Further studies should employ more objective data to measure central adiposity and explore its associations with ANM.

Our study found that clinically diagnosed hypertension was positively associated with early menopause, which was similar to some previous studies [46,47]. A potential explanation is that hypertension could cause follicle loss and ovarian reserve reduction, which would accelerate the onset of menopause [48]. The mechanism underlying hypertension and ANM is not fully clarified [49]. It is unclear whether hypertension accelerates menopause, resulting in earlier menopause, or if it is a natural phenomenon of aging or results in early menopause. In another Chinese study, Wang et al. inversely found that clinical hypertension was associated with late menopause [22]. This inconsistency suggests that further efforts are needed to explore the association between hypertension and ANM.

Interestingly, our study found that hyperlipidemia was positively associated with late menopause. The potential mechanism behind the association between ANM and hyperlipidemia could be related to serum total cholesterol (TC), an indicator of hyperlipidemia [50]. However, current studies present conflicting evidence on the association between serum TC and ANM. Several studies have reported that they did not observe any significant association between serum TC and ANM [51–53], whereas data from the Framingham Heart Study cohort suggest that higher premenopausal serum TC was related to early menopause [54]. The present study only revealed that late menopause was associated with higher serum TC in postmenopausal women. Given this, the relationship between hyperlipidemia and ANM remains unclear, necessitating further exploration [55,56].

Our study had some strengths. It was based on a large sample size survey obtained by sampling the distribution of women in corresponding age groups across 11 cities in Zhejiang Province. The sampling method employed in this study was designed to yield a representative sample of women within the selected age groups, enhancing the generalizability of our findings concerning the ANM to other women in this age group residing within the province. Additionally, the large sample size of our study likely ensure sufficient statistical power to detect even modest yet meaningful associations as statistically significant.

There are also several limitations. Firstly, although our sample included participants from rural areas, it may not accurately represent the ANM and associated factors for women from economically disadvantaged areas in China. Secondly, there were some problems with independent variables. For instance, our study did not collect data on high levels of physical exercise. We just explored the association between regular physical exercise and ANM. Thirdly, the cross-sectional design of our study did not allow the assessment of the temporal relationships among variables like central adiposity, hypertension, and hyperlipidemia. Further research should employ a longitudinal study design to explore the associations between these factors and the ANM. Specifically, it is crucial to examine the associations between the duration of these conditions and ANM. Also, the uncertain regarding whether the data on smoking and physical activity represent current or pre-menopausal status led to the possibility of reverse causality. Fourthly, there was the potential for misclassification and non-comparability of data due to the use of self-reported WC, hypertension, diabetes, and hyperlipidemia and the lack of standard instruments for data collection, rather than objective measurements. Additionally, it was unclear whether the self-reported conditions were diagnosed prior to or coinciding with the onset of menopause. This temporal uncertainty could significantly impact the interpretation of the associations observed. Furthermore, if these conditions were diagnosed before the ANM, it is conceivable that subsequent pharmacological interventions could have influenced the ANM. Such interventions might potentially confound the causal relationship between these health conditions and ANM, thereby obscuring their association. Next, we did not examine any effect modifications or interactions, which may lead to an incomplete understanding of the complex relationships among the variables under study. Additionally, our study was only concerned with natural menopause. However, artificial menopause and associated factors also need to be attention. Meanwhile, we just explored the associated factors with ANM. Postmenopausal risks with early menopause and late menopause still need further examination. Finally, our study was conducted during the COVID-19 pandemic, which could have potentially influenced our survey results.

## Conclusion

We found the median ANM of women in our study from Zhejiang was 51 years. This study identified the associated factors with early and late menopause including social demographics, lifestyle behaviors, reproductive history, and health-related factors. The results suggest that social demographic factors including age, life behaviors factors including current or prior smoking status, reproductive history factors including irregular menstrual cycles, miscarriages, and breastfeeding, and health-related factors including central adiposity, hypertension, and hyperlipidemia, were associated with the timing of natural menopause. Women who experience early menopause and late menopause probably face many long-term adverse health outcomes. It is important to identify the factors related to ANM. These findings provide a basis for understanding various factors associated with ANM.
